# Treat-to-Target in Lupus Nephritis. What is the Role of the Repeat Kidney Biopsy?

**DOI:** 10.1007/s00005-022-00646-9

**Published:** 2022-02-11

**Authors:** Ioannis Parodis, Farah Tamirou, Frédéric A. Houssiau

**Affiliations:** 1 Division of Rheumatology, Department of Medicine Solna, Karolinska Institutet, and Karolinska University Hospital, 171 76 Stockholm, Sweden; 2 Department of Rheumatology, Faculty of Medicine and Health, Örebro University, Örebro, Sweden; 3 Pôle de Pathologies Rhumatismales Inflammatoires et Systémiques, Institut de Recherche Expérimentale et Clinique, Université Catholique de Louvain, Brussels, Belgium; 4 Rheumatology Department, Cliniques Universitaires Saint-Luc, Brussels, Belgium

**Keywords:** Systemic lupus erythematosus, Lupus nephritis, Treat-to-target, Kidney biopsy, Kidney disorders, Autoimmunity

## Abstract

Kidney involvement, termed lupus nephritis (LN), develops in 35–60% of patients with systemic lupus erythematosus, often early during the disease course. When not treated promptly and efficiently, LN may lead to rapid and severe loss of kidney function, being the reason why it is considered one of the most severe lupus manifestations. Despite improved pharmacotherapy, 5–20% of LN patients develop end-stage kidney disease within ten years from the LN diagnosis. While the principal ground of LN therapy is prevention of renal function worsening, resembling a race against nephron loss, consensual agreement upon outcome measures and clinically meaningful short- and long-term targets of LN therapy have yet to be determined. Literature points to the importance of inclusion of tissue-based approaches in the determination of those targets, and evidence accumulates regarding the importance of per-protocol repeat kidney biopsies in the evaluation of the initial phase of therapy and prediction of long-term renal prognosis. The latter leads to the hypothesis that the information gleaned from repeat biopsies may contribute to optimised therapeutic decision making, and, therefore, increased probability to attain complete renal response in the short term, and a more favourable renal prognosis within a longer prospect. The multinational project ReBioLup was recently designed to serve as a key contributor to form evidence about the role of per-protocol repeat biopsies in a randomised fashion and aspires to unify the global LN community towards improved kidney and patient survival.

## Introduction

Systemic lupus erythematosus (SLE) is a chronic autoimmune disease that is characterised by a broad spectrum of organ manifestations, and a broad range of degree of severity (Kaul et al. 2016). Kidney involvement develops in 35–60% of patients with SLE, often early during the disease course (Anders et al. 2020; Cervera et al. 2003; Pons-Estel et al. 2011; Singh and Saxena 2009). When not treated promptly and efficiently, lupus nephritis (LN) may lead to rapid and severe loss of kidney function, being the reason why it is considered one of the most severe manifestations of SLE. Even today, despite improved pharmacological and non-pharmacological management, 5–20% of LN patients develop end-stage kidney disease (ESKD) within ten years from the LN diagnosis (Anders et al. 2020; Houssiau et al. 2004, 2010; Pons-Estel et al. 2011; Tektonidou et al. 2016; Vandepapeliere et al. 2014; Zhang et al. 2016).

Indeed, recent trial successes resulted in approval by regulatory agencies of the two first in history drugs for LN, i.e., the new generation calcineurin inhibitor voclosporin (Rovin et al. 2019, 2021) and the monoclonal antibody against B cell activating factor (BAFF; also known as B lymphocyte stimulator, BLyS) belimumab (Furie et al. 2020), as add-on treatments on top of standard disease modifying therapy (Fanouriakis et al. 2020). With regard to the pipeline, the fully humanised B cell depleting anti-CD20 obinutuzumab (Furie et al. 2021) and the fully human anti-type I interferon receptor (IFNAR) anifrolumab (Jayne et al. 2021) are currently trialled, and results from interim data analyses are encouraging. Still, the divergent primary and key secondary endpoints or even diverse components in definitions of complete and partial renal response across the trials illustrate the lack of consensual agreement upon the goals of therapy, and the overall low percentages of responding patients not reaching half of the study participants, even among those given the active substance, is indicative of the fact that there still is room for further improvement (Parodis and Houssiau 2021). Towards this overarching goal, granular understanding of the disease pathophysiology, employment of tissue-based approaches to determine therapeutic targets, and identification of non-invasive biomarkers of renal activity and response to therapy are imperative.

## LN Facets and Choice of Therapy

Mechanistically, the pathogenesis of LN is multifactorial, with inflammatory responses to immunogenic, endogenous chromatin being a key contributor. Such nuclear material, which is abundant in patients with SLE, activates sensors such as Tool-like receptor (TLR)7 and TLR9 in innate immune cells, triggering type I interferon (IFN) production and production of pro-inflammatory cytokines. B cells are persistently activated in SLE, and autoantibody production is perpetual, with mediators such as BAFF precipitating this milieu. T cells also have key roles, e.g., T follicular helper cells and interleukin (IL)-17 producing T helper 17 cells are known to drive kidney injury (Anders et al. 2020; Parodis et al. 2015; Zickert et al. 2015).

Autoreactive leukocytes, immune complexes (IC) and various susceptibility genes are involved in renal injury, mediated by cytokines, chemokines, and growth factors. Complement proteins are also involved, with active SLE-related renal injury often resulting in reduced circulating complement levels. Molecules that have been targeted in drug development research for LN include BAFF, B cell surface markers (e.g., CD20 and CD22), immune co-stimulatory molecules, cytokines (IL-6, IFN-γ, IFN-α) and the IFN-α/β receptor (IFNAR) (Anders et al. 2020). As a matter of course, the pronounced biological heterogeneity in LN poses therapeutic challenges, necessitating individualised approaches. Nevertheless, whatever the choice of treatment, the long-term treatment target is always the same, i.e., preservation of the kidney function, which also forms the basis for shorter-term treatment goals.

The severity of LN and thus the choice of treatment is however not only determined by the IC-mediated renal injury, but also multiple other factors with pathogenic contributions. Such factors include mechanistic facets such as podocytopathy, hyperfiltration and proteinuria, the latter constituting the cardinal clinical symptom of LN, as well as extra-renal disease, renal and extra-renal toxicity resulting from immunosuppressants, and associated comorbidities. Patients with LN have a high risk to develop a wide range of comorbid conditions, such as cardiovascular disease and treatment-related adverse events, e.g., infections, ovarian failure and fertility issues, and osteoporosis (Anders et al. 2020). Obesity and diabetes are comorbidities of particular importance for patients with LN due to their nephropathogenicity, making surveillance for these factors and encouragement of lifestyle modifications to improve patients’ lipidaemic and glycaemic profiles imperative for all SLE patients with renal involvement (Houssiau 2012). Adjunct therapy along with immunosuppression is therefore crucial; this should always include antiproteinuric and antihypertensive blockade of the renin–angiotensin–aldosterone system in non-pregnant patients. Non-steroidal anti-inflammatory drugs should be avoided, and the need for statin therapy should be accounted for. Primary and secondary prevention of thrombosis is important in the presence of a high-risk antiphospholipid profile, and bone protection should follow both pharmacological and non-pharmacological management, e.g., exercise and weight control. Last but not least, antiplatelet or anticoagulant therapy is important in patients with histopathological findings consistent with antiphospholipid syndrome nephropathy, e.g., acute thrombotic microangiopathy (Fanouriakis et al. 2020).

## Goals of LN Therapy

Based on the 2019 update of the joint European League Against Rheumatism and European Renal Association—European Dialysis and Transplant Association (EULAR/ERA-EDTA) recommendations for the management of lupus nephritis, the therapy for LN should aim for a proteinuria of 0.5–0.7 g/day or less by month 12 from baseline, along with control of the extra-renal disease. An adequate proteinuria decrease is ≥ 50% within six months from baseline, or up to one year for patients with nephrotic-range baseline proteinuria (Fanouriakis et al. 2020). Robust evidence that early decrease in proteinuria predicts good long-term renal outcome comes from recent studies, including analyses of data from the Euro-Lupus Nephritis Trial (Houssiau et al. 2010) and the MAINTAIN Nephritis Trial (Tamirou et al. 2016). Three independent studies indicated a cut-off of 0.7–0.8 g/day at one year of treatment to be the best predictor of favourable long-term renal outcome, defined as a creatinine value ≤ 1 mg/dL seven years after the LN onset (Dall'Era et al. 2015; Tamirou et al. 2015; Ugolini-Lopes et al. 2017). However, reliable predictors of poor long-term prognosis have yet to be determined, shepherding the attention to the gold standard, i.e., the information gleaned from the renal tissue.

Kidney function declines with age as a consequence of gradual nephron loss, with an average kidney function lifespan of 120 years, or lower in individuals born with fewer nephrons or sufferers from acute or chronic kidney disease. Each LN flare results in irreversible nephron loss, substantially reducing the lifespan of the kidneys by years or decades. When LN remains persistently active, the rate of nephron loss increases, subsequently resulting in earlier onset of ESKD (Anders et al. 2020). Thus, renal relapses and persistent activity constitute predictors of renal function impairment and development of ESKD, and short-term goals of LN treatment should include induction and maintenance of complete renal response to prevent new flares and further nephron loss.

Importantly, patients with LN suffer a substantially impaired quality of life compared with the general population, which is not only as a consequence of the renal injury, but also side-effects of immunosuppressant and glucocorticoid use, as well as the socioeconomic disease burden (Anders et al. 2020). Pharmacological and non-pharmacological management of LN should therefore also aim for amelioration of physical, mental, and social aspects of the patients’ quality of life.

## Repeat Kidney Biopsy

The baseline kidney biopsy is indispensable for the diagnosis and classification of LN since markers which reliably reflect kidney histopathology are lacking, although current classification (Churg et al. 1995; Weening et al. 2004) needs refinements to further stress on the tubulointerstitial compartment (Bajema et al. 2018) and include determination of prognostic histological patterns, e.g., patterns that are associated with poor long-term renal outcome. For the latter, artificial intelligence and machine learning may play important roles in future endeavours. The role of repeat kidney biopsy has been debated for decades, and achievement of consensus is, albeit desirable, still pending. One of the hurdles has been the discrepancies in how the term “repeat biopsy” has been used in literature. Hans-Joachim Anders recently summarised clinical scenarios in which repeat biopsies have been performed, and described the definition that the term was designated in each one of those scenarios (Anders 2018).

The first scenario was that of the per-protocol repeat biopsy with the purpose of evaluating the initial phase of immunosuppressive therapy for active LN, also termed induction phase, and deciding the therapy to be given thereafter. A second scenario was that of partial response where a distinction between residual renal activity and delayed healing process has to be made to adjust the degree of immunosuppression accordingly. Repeat kidney biopsy when there is a suspicion of flare and biopsy after a period of quiescent renal disease to support alleviation or withdrawal of the immunosuppressive therapy also constitute possible scenarios. Finally, repeat biopsies sometimes are performed to distinguish between irreversible chronic damage, e.g., glomerular sclerosis, tubular atrophy or chronic vascular changes, and treatable active injury in cases of progression of the degree of renal insufficiency.

In studies of repeat kidney biopsies performed to evaluate treatment outcome after the initial phase of immunosuppression, the discordance between clinical and histopathological evaluation has been striking. In the concrete, residual active lesions appear in biopsies from a substantial proportion of patients who have displayed complete clinical response to treatment assessed by the level of proteinuria, serum creatinine concentrations and findings in the urinary sediment (Arends et al. 2012; De Rosa et al. 2018; Hill et al. 2000; Malvar et al. 2017; Pineiro et al. 2016; Zickert et al. 2014).

## Predictors of Renal Outcome: What Do Repeat Biopsies Tell?

Early decrease in proteinuria (Houssiau et al. 2010; Korbet et al. 2013; Tamirou et al. 2016) and proteinuria levels < 0.7–0.8 g/day one year after treatment initiation are robust early markers with ability to predict favourable long-term renal outcome (Dall'Era et al. 2015; Korbet et al. 2013; Tamirou et al. 2015; Ugolini-Lopes et al. 2017). However, several studies have provided strong implications of discrepant patterns between the clinical outcome of therapy, mainly determined by proteinuria, and the histopathological outcome, which is based on activity and chronicity features in repeat kidney biopsies, e.g., demonstrating persistent treatable renal activity at the level of tissue despite complete clinical response (Arends et al. 2012; De Rosa et al. 2018; Malvar et al. 2017; Pineiro et al. 2016; Zickert et al. 2014). Predictive markers of adverse long-term prognosis have been lacking, with the possible exception of early chronic tissue damage, that has been implied as a predictor of poor prognosis since the early 1980’s in a work by Austin et al. (1983). Later studies, however, including a recent investigation by our group, could not confirm that chronic changes in the initial biopsy are indicative of the long-term prognosis (Parodis et al. 2020a; Schwartz et al. 1989).

As mentioned above, the ultimate target of the therapeutic management of LN is prevention of impairment of the renal function in the long prospect. Since renal flares contribute to nephron and renal function loss, prevention of flares may be considered a major short-term target. Clinical and translational research in the field of LN has sought to identify early clinical, laboratory or molecular markers that are coupled with poor long-term renal outcome, aiming for improvement in the monitoring and overall management of patients with LN. Genes, initial nephron cargo, global SLE course and drug-mediated nephrotoxicity are some of the factors that have been shown to contribute to renal function impairment, with renal flares accounting for a major proportion of nephron loss (Anders et al. 2020). Importantly, current therapies induce complete renal remission in a relatively small percentage of LN cases (Appel et al. 2009; Ginzler et al. 2005), illustrating the unmet need for better therapeutics. Collectively, it appears paramount to determine early predictors of long-term renal prognosis, and use this information to improve the management of patients with LN.

We have called attention to the potential role of per-protocol repeat kidney biopsies to evaluate the treatment outcome and guide subsequent therapeutic decision-making (Parodis et al. 2020c). Indeed, while the importance of an initial diagnostic biopsy to determine type of nephritis and exclude mimickers is indisputable (Bihl et al. 2006; Fanouriakis et al. 2020; Hahn et al. 2012), repeat kidney biopsies after the incident LN episode appear to bear a more informative message with regard to long-term renal prognosis. In a recent retrospective study of incident proliferative LN, with or without a concurrent membranous histological pattern, information gleaned from per-protocol repeat kidney biopsies showed ability to predict short- and long-term renal outcomes, i.e., subsequent renal relapses and impairment of the renal function, respectively (Parodis et al. 2020a). More specifically, NIH activity index scores > 3 in the repeat kidney biopsies predicted subsequent renal flares, whereas NIH chronicity index scores > 3 in the repeat biopsies were associated with renal function deterioration in the long term. It is worth noting that active lesions in glomeruli mostly accounted for the former association with renal relapse, whereas chronic damage in the tubulointerstitial compartment was found to be an important contributor to the latter association with long-term renal function (Parodis et al. 2020a). This observation is of particular importance in light of the fact that tubulointerstitial injury and damage have been rather neglected in current LN classification (Table 1) (Weening et al. 2004), although the contribution of the tubulointerstitial compartment of the kidney in the inflammatory process in LN (Bonanni et al. 2015; Eddy 2004; Ronda et al. 2005; Theilig 2010; Yap et al. 2016; Zheng et al. 2008) and its importance in renal prognosis (Broder et al. 2018; Clark et al. 2015; Daniel et al. 2001; Ferraccioli and Romano 2008; Hsieh et al. 2011; Yu et al. 2010) has been extensively advocated for in literature.

**Table 1:** The 2003 ISN/RPS classification of lupus nephritis

Class	Definition
I	Minimal mesangial LN
II	Mesangial proliferative LN
III (A)	Focal proliferative LN (active lesions)
III (A/C)	Focal proliferative and sclerosing LN (active and chronic lesions)
III (C)	Focal sclerosing LN (chronic inactive lesions with glomerular scars)
IV-S (A)	Diffuse segmental proliferative LN (active lesions)
IV-G (A)	Diffuse global proliferative LN (active lesions)
IV-S (A/C)	Diffuse segmental proliferative and sclerosing LN (active and chronic lesions)
IV-G (A/C)	Diffuse global proliferative and sclerosing LN (active and chronic lesions)
IV-S (C)	Diffuse segmental sclerosing LN (chronic inactive lesions with scars)
IV-G (C)	Diffuse global sclerosing LN (chronic inactive lesions with scars)
V	Membranous LN
VI	Advanced sclerosing LN

*ISN* International Society of Nephrology, *RPS* Renal Pathology Society

## A Study to Form Evidence for Per-Protocol Repeat Biopsy

**Fig. 1:**
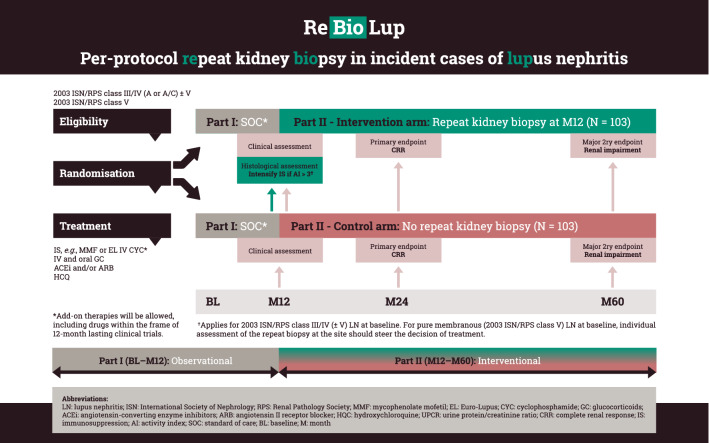
Flowchart of ReBioLup

Collectively, evidence supporting the importance of repeat kidney biopsies as an integral part of the evaluation of the initial phase of treatment for LN accumulates. A joint effort has therefore emerged within the frame of the Lupus Nephritis Trials Network to design a prospective study, with the aim to investigate whether the histopathological information obtained from per-protocol repeat kidney biopsies 12 months after initiation of immunosuppression for incident biopsy-ascertained active LN results in treatment changes which in turn favour long-term renal outcome, contributing to preservation of the renal function. The plan is to soon embark upon this multinational endeavour, which is titled “Per-protocol repeat kidney biopsy in incident cases of lupus nephritis”, or shortly ReBioLup (http://rebiolup.com). Within the frame of this project, study participants will be randomised to either undergo or not undergo a per-protocol repeat kidney biopsy one year after the initial biopsy and initiation of treatment, as illustrated in the ReBioLup flowchart in Fig. 1. This design will enable us to determine whether therapeutic adjustments based on the results of the repeat kidney biopsy, and more specifically intensification of the immunosuppressive therapy in cases of residual activity at the level of tissue, contribute to better renal prognosis compared with standard of care approaches in the control group of patients who will not undergo repeat biopsy, i.e., blinded to tissue-level disease activity. In brief, patients with a 2003 ISN/RPS class III or IV (± V) LN and an activity index score > 3 in the repeat kidney biopsy will receive intensified immunosuppressive treatment according to the physician’s and patient’s shared decision, whereas all other patients will be treated according to current recommendations (Fanouriakis et al. 2020). For 2003 ISN/RPS class V (pure membranous) LN where data to support such guidance are lacking, individual assessment of the repeat kidney biopsy will dictate subsequent management, and results from ReBioLup are expected to inform the LN researcher community towards optimised future strategies.

The design of ReBioLup has been based on data from studies of proliferative LN, since current knowledge about membranous LN is inadequate. However, patients with a biopsy-proven active membranous LN will be asked to participate, and one of the main goals with the project will be to increase the understanding of this LN subtype. Reabsorption of subepithelial and intramembranous electron dense deposits after therapy with cyclophosphamide and rituximab in a combined regimen has been shown to be coupled with long-lasting clinical improvement, including reductions in proteinuria levels (Jonsdottir et al. 2011), possibly with distinct patterns across treatment regimens (Zickert et al. 2021). Within the frame of ReBioLup, electron microscopy will be performed to assess the renal immunological response to treatment, i.e., changes in electron dense deposits, in contrast to histopathological changes alone which currently are the common features used to evaluate tissue-level outcomes. We strongly believe that the immunological changes following treatment carry important information for therapeutic decision-making, and assessment of electron micrographs might change the state of the art in kidney biopsy evaluation, particularly with regard to membranous LN.

An important strength of ReBioLup is its observational design during the initial phase of therapy, aiming for pragmatic requirements to ensure feasibility and data that derive from real-life clinical scenarios. The prospective comparison with a control arm is novel in a setting of systematically performed repeat biopsies, and the combination of clinical, histopathological, serological and urinary data from the same sampling occasions will provide a unique opportunity of integrated analysis to determine reliable non-invasive biomarkers that reflect immune-mediated inflammatory activity and chronic irreversible damage at the level of tissue, as well as non-invasive markers with value in prognostication of the long-term renal outcome. Ultimately, the goal is to substitute the kidney biopsy with less invasive methods for assessment of activity and chronicity as well as prognostication, and it is heartening that several efforts in this direction have been inaugurated, e.g., studies of urinary biomarkers, in several of which CD163 (Gupta et al. 2021; Mejia-Vilet et al. 2020; Zhang et al. 2020) and the activated leukocyte cell adhesion molecule (Ding et al. 2020; Parodis et al. 2020b; Stanley et al. 2020; Chalmers et al. 2022) have emerged as examples of promising markers that reflect renal histology. In this respect, investigations of how non-invasive biomarkers relate to renal histopathology in prospective endeavours such as ReBioLup are eagerly awaited.

## Concluding Remark

To summarise, whereas the management of patients with LN has advanced with the introduction of new therapeutic modalities, there is still an imperative need for improvement to prevent loss of renal function and development of ESKD in the long prospect of the course of SLE patients with kidney affliction. Towards this goal, consensual outcome measures and clinically meaningful short- and long-term targets of LN therapy need to be determined and include tissue-based information. Such needs call for unified efforts in international endeavours. ReBioLup is designed to serve as a key contributor in this direction and aspires to unify the global lupus nephritis community towards improved kidney and patient survival, and preservation of the renal function in this complex SLE patient subgroup.
